# Case of Universal Tuberculosis

**Published:** 1860-09

**Authors:** I. P. Kluge

**Affiliations:** Physician to the Panama Railroad Company on the Isthmus, and formerly one of the Resident Physicians at the Emigrants’ Refuge Hospital, New York


ART. VII. — *Case of Universal Tuberculosis.* Reported by I. P. KLUGE, M.D., Physician to the Panama Railroad Company on the Isthmus, and formerly one of the Resident Physicians at the Emigrants' Refuge Hospital, New York.

MARIA SUSS, native of Germany, aged twenty-three, died February 8th, 1854, at the Emigrants' Hospital, in New York. The patient was admitted into Ward 26 of this hospital, about one month ago, complaining at the time only of suppression of the menses. Shortly after her admission, however, symptoms of a latent and more serious disease developed themselves. She became more and more emaciated from day to day, complained of a dry, hacking cough, which, at the time of her admission, and prior to that, she had not been troubled with. Upon a careful physical examination, I became satisfied that both lungs were infiltrated with tubercles, although the sputa and auscultation did not indicate any great degree of softening. The treatment adopted was that usually employed in tuberculous and strumous cases—nutritious diet, stimulants, tonics, ol. jecoris aselli, and phosphate of lime.

The patient sank so suddenly and rapidly, notwithstanding all treatment, that both my colleague, Dr. Rothe, and myself came to the conclusion that the case was one of acute phthisis, and, probably, of universal tuberculosis also. There was no diarrhœa at any time. The patient's skin was very dry, and unusually pale and shriveled.

An autopsy was made February ninth, twenty hours after death.

The appearance of the heart was pale and bloodless, but there was no evidence of organic disease; the pericardium was distended with fluid, and upon being opened was found to contain four ounces of limpid, straw-colored serum; the left lung was greatly atrophied and infiltrated with tubercles throughout its whole extent—some of these were crude, others had become calcareous. There was œdema of the inferior lobe of this lung, and old pleuritic effusion on right lung, bands of lymph connecting the pleura pulmonalis firmly with the pleura costalis. The right lung was also filled with masses of tubercle, and there was œdema of the inferior half. The liver was much enlarged, very pale, and almost white; and had undergone fatty degeneration, as shown by the appearance presented by the scalpel when drawn through its substance, as well as by the paper test. The mesenteric glands also were much increased in size, and as large as pigeons' eggs, and indurated. A glandular mass of much the same appearance and consistence, and of the size of an almond, was discovered on the external surface of the pericardium. The mesentery itself, omentum, and the external surface of a great portion of the small intestines, were studded with small, granular tubercles, forming prominences and elevations. These latter phenomena I had never observed in any previous post-mortem of a case of tuberculosis, and believe them to be of by no means common occurrence.

The spleen was much congested, of a bright, cherry-red color, and filled at intervals of one and two inches with large tubercles. The peritoneal covering and broad ligaments of the uterus were also filled with tubercles, but none could be detected in either the substance of the uterus or ovaries; the latter were very pale and anæmic. The woman must have had leucorrhœa, which she did not state, as was apparent from the condition of the cervix uteri and the mucus secreted in it and about the os. The uterus was considerably indurated; its general appearance, however, was not that of carcinoma, although when the knife was drawn through its tissues the peculiar grating sensation was communicated. Serous cysts, resembling gelatinous polypi or hydatids, of the size of an ordinary walnut, were found adherent to the external coat of the small intestines, near their junction with the mesentery.

Both kidneys were normal; the pancreas was enlarged, indurated, and adherent to the pyloric portion of the stomach; the mucous membrane of the stomach was much congested, which accounted for the patient's inability to retain solid food, as well as the acute pain she latterly experienced in the gastric region. The mucous coat of the duodenum had undergone great softening, and could easily be scraped off with the handle of the scalpel, though there was no ulceration or other abnormal condition of the mucous surface of other portions of the alimentary canal observable. The brain and spinal marrow were not examined.

